# The Neural Substrates Underlying the Implementation of Phonological Rule in Lexical Tone Production: An fMRI Study of the Tone 3 Sandhi Phenomenon in Mandarin Chinese

**DOI:** 10.1371/journal.pone.0159835

**Published:** 2016-07-25

**Authors:** Claire H. C. Chang, Wen-Jui Kuo

**Affiliations:** 1 Institute of Neuroscience, National Yang-Ming University, Taipei, Taiwan; 2 College of Humanities and Social Sciences, Taipei Medical University, Taipei, Taiwan; 3 Brain Research Center, National Yang-Ming University, Taipei, Taiwan; Sun Yat-sen University, CHINA

## Abstract

This study examined the neural substrates underlying the implementation of phonological rule in lexical tone by the Tone 3 sandhi phenomenon in Mandarin Chinese. Tone 3 sandhi is traditionally described as the substitution of Tone 3 with Tone 2 when followed by another Tone 3 (33 →23) during speech production. Tone 3 sandhi enables the examination of tone processing in the phonological level with the least involvement of segments. Using the fMRI technique, we measured brain activations corresponding to the monosyllable and disyllable sequences of the four Chinese lexical tones, while manipulating the requirement on overt oral response. The application of Tone 3 sandhi to disyllable sequence of Tone 3 was confirmed by our behavioral results. Larger brain responses to overtly produced disyllable Tone 3 (33 > 11, 22, and 44) were found in right posterior IFG by both whole-brain and ROI analyses. We suggest that the right IFG was responsible for the processing of Tone 3 sandhi. Intense temporo-frontal interaction is needed in speech production for self-monitoring. The involvement of the right IFG in tone production might result from its interaction with the right auditory cortex, which is known to specialize in pitch. Future studies using tools with better temporal resolutions are needed to illuminate the dynamic interaction between the right inferior frontal regions and the left-lateralized language network in tone languages.

## Introduction

Human languages could be divided into two broad categories according to whether they use pitch patterns to distinguish words or the grammatical forms of words. Those who don’t are non-tone languages. Those who do are tone languages and pitch patterns they used are called lexical tones. For example, in Mandarin Chinese, the syllable /ma/ could mean “mother” when pronounced with a high level tone (Tone 1), or “horse” with a falling-rising tone (Tone 3). According to UPSID [1], a database sampled existent languages based on genetic diversity, tone languages not only account for at least 40% of the languages in the world but are also geographically widely distributed, including most part of Africa, the east and southeast Asia, and some regions in America [2,3]. In other words, the usage of lexical tone is a common practice in human languages rather than exceptional or deviant. However, most of our knowledge about the language processing network is based on studies in non-tone languages. In terms of phonological processing, language models built on these studies [4–6] mainly focus on segments, e.g. consonant and vowel, but ignore tone. Segment undoubtedly is more prevalent than tone. It serves as important phonological unit in tone language as well as in non-tone languages. But, as illustrated above, tone also plays an essential role in human languages. Most importantly, tone has acoustic and articulatory properties distinct from segment, which, as the next section will show, lead to neural processing different from segment. Therefore, the incorporation of tone in models of language processing by investigating tone languages is indispensable.

Based on findings in pitch without linguistic function (referred to as non-linguistic pitch in the remaining part of this study)[7–11], a functional asymmetry between the left and right auditory cortices has been proposed. Zatorre [11] suggested that the left auditory areas has better temporal resolution, while the right auditory areas has better spectral resolution. The asymmetric sampling in time hypothesis (AST), on the other hand, proposed that the left auditory areas extract information from short (~20–40 ms) temporal integration windows, while the right auditory areas extract from information from long (~150–250 ms) integration windows [7]. According to both hypotheses, since tone has richer spectral information and longer duration, tone processing should rely more on the right auditory cortex. Indeed, compared to segment, the perception of lexical tone elicited more activations in the right auditory cortex [12–16]. Further, the right anterior superior temporal gyrus (STG) was found to be functionally connected with the left-lateralized language network for the comprehension of Chinese, but not English [17], implying that the integration of information from the left and right auditory areas is crucial for the comprehension of tone languages.

Most previous works in tone processing have focused on perception [18], while tone production remains relatively unexplored. This study aims to investigate the neural networks of lexical tone production. Specifically, we examined the neural substrates underlying the implementation of phonological rule in lexical tone by the Tone 3 sandhi phenomenon in Mandarin Chinese. There are four lexical tones in Mandarin Chinese. Tone 3 sandhi is traditionally described as the substitution of Tone 3 with Tone 2 when followed by another Tone 3 [19]—i.e., underlying tone sequence 33 is pronounced as 23 on the surface. Tone 3 sandhi is a phonological rule similar to the a/an alternation in English (an apple vs. a dog), only that it operates on tone rather than segment. Tone 3 sandhi automatically changes the tone representation regardless of the concurrent segments [19–21], thus provides a chance to explore the processing of tone in a more abstract level with the least involvement of segments. It is important to recognize that tone sandhi is not specific to Mandarin Chinese, sandhi rules have been widely found in tone languages across Africa and Asia [22,23]. One tone language could have various sandhi rules. Disregard the surface diversity, there could be some general neural mechanisms underlying tone sandhi rules and this study used the Tone 3 sandhi phenomenon in Mandarin Chinese as a probe.

Where would the tone sandhi rule be implemented in the brain? Loui et al. [24] has created a pitch-based artificial grammar and found that the participants’ learning performance positively correlated with the volumes of the right arcuate fasciculus connecting the right inferior frontal gyrus (IFG) and the superior temporal lobe, which implies that the right inferior frontal regions are involved in higher order pitch processing through temporo-frontal interaction [25,26]. If this is true even for pitch with linguistic function, the right inferior frontal regions and the right arcuate fasciculus should also play a role in the processing tone languages. Indeed, the integrity of the indirect pathway of the arcuate fasciculus [27] has been reported to predict English speakers' performances in learning Mandarin Chinese [28]. According to the dual stream model of speech processing [29], the dorsal temporo-frontal pathway via arcuate fasciculus is for the sound-to-articulation mapping, while the ventral pathway is more for the sound-to-meaning mapping. It is worth noticing that the integrity of the left ventral pathway via the extreme capsule and the inferior longitudinal fasciculus has been reported to be correlated with English speakers’ performance in learning the association between tonal syllables and word meanings [30].

One of our previous fMRI experiments provides primary evidences for the involvement of right inferior frontal regions in Tone 3 sandhi processing [31]. We examined the brain activations corresponding to the production of several four-syllable tone sequences. Compared to other repeated sequences (1111, 2222, and 4444), sequence 3333, which triggered Tone 3 sandhi, elicited right-lateralized activations in anterior insula and pIFG. However, since we did not include monosyllable tones in the previous study, whether our findings reflected the processing of Tone 3 itself or the processing of Tone 3 sandhi remains debatable. If monosyllable Tone 3, which does not trigger Tone 3 sandhi, still elicits similar brain responses, then our findings could not be the results of Tone 3 sandhi, but more likely to reflect the physical difficulty of producing Tone 3. Tone 3 might be physically harder to pronounce because it has the most complicate contour (falling-rising) among the four Mandarin lexical tones, at least in standard Mandarin.

In this study, we asked the participants to pronounce monosyllable and disyllable sequences of tones. Since Tone 3 sandhi only applies to sequence of Tone 3, we expected that the contrast between Tone 3 and other tones to reveal brain responses associated with Tone 3 sandhi only under the disyllable condition (33 > 11, 22, and 44), but not the monosyllable condition. In addition, we also tried to distinguish the pre-articulatory planning stage and the motor execution stage of speech production by manipulating the requirement on overt oral response [25,32–34]. Speech production is often divided into two stages [5,35–39]. The pre-articulatory planning stage includes retrieval and sequencing of abstract phonological representations, while the execution stage is responsible for the coordination and control of individual articulators, e.g. tongue and lip. We predicted that the Tone 3 sandhi effect would either occur both with and without overt oral response or occur only when overt response was executed. The former outcome would support that Tone 3 sandhi is processed at the pre-articulatory planning stage, while the later would suggest that Tone 3 sandhi is dependent on motor execution.

## Materials and methods

### Participants

Thirty college students who were right-handed, native Taiwan Mandarin speakers with no history of neurological disorders and normal or corrected-to-normal vision were recruited. Six of them were excluded from further imaging and acoustic analyses due to high error rate or not showing Tone 3 sandhi (see sound recording analysis section for details). The average age of the remaining twenty-four participants is twenty-four, including fourteen females. Written consent was obtained before MR scanning, with the protocol approved by the Institutional Review Board of National Yang-Ming University.

### Materials and procedure

A 4 x 2 x 2 design was used, with the following factors: tone (1, 2, 3, and 4), number of syllable (monosyllable and disyllable), and overt oral response (overt or no oral response). We expected to reveal brain responses associated with Tone 3 sandhi processing only under the disyllable condition with the contrast between Tone 3 and other tones (33 > 11, 22, and 44) and were interested in whether such effect would be modulated by overt oral response.

**Fig 1:**
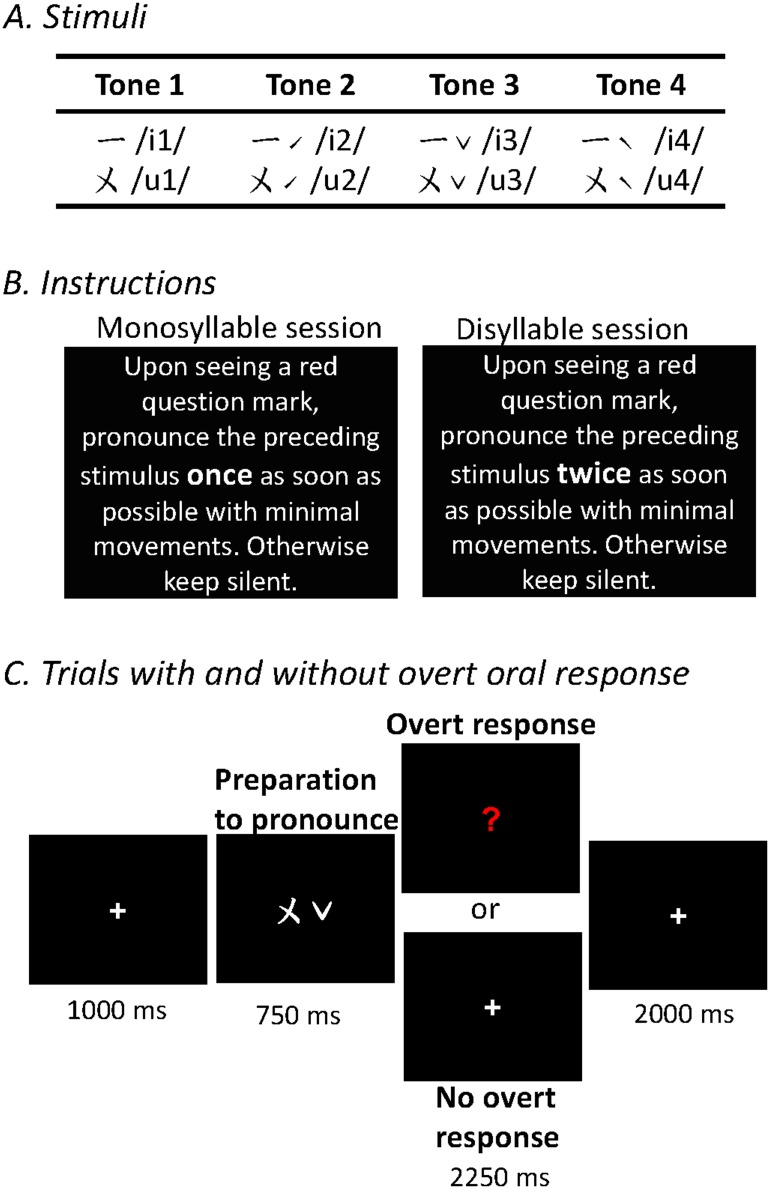
Experimental stimuli and procedure. We manipulated tone, number of syllable, and the execution of oral response. Row A, the stimuli for the four lexical tones presented in Chinese phonetic symbols, Zuyin. Their corresponding international phonetic alphabets are enclosed by slashes. The symbol on the left side denotes the vowel, while the right one denotes the tone. Tone 1 is denoted by the absence of tonal symbol. Row B, the instruction for the monosyllable and disyllable sessions. Row C, the procedures of trials with and without overt oral response.

The four lexical tones in Mandarin Chinese were combined with vowel /i/ and /u/ to generate eight monosyllables as the stimuli. They were visually presented in a Chinese phonetic marking system, Zuyin (Fig 1, row A). The participants were asked to pronounce the stimuli upon seeing a following response cue, which occurred in half of the trials, and keep silent otherwise (Fig 1, row B and C). The participants were instructed to pronounce the monosyllable stimuli once in two scanning sessions, twice in the other two sessions (Fig 1, row B). The order of the monosyllable and disyllable sessions was counterbalanced across participants.

Each session comprised eighty experimental trials, ten for each stimulus, and twenty baseline trials, in which unpronounceable pseudo-characters were presented. Overt oral response was only executed in half of the experimental trials. These trials were randomly ordered and each lasted for 6,000 ms, starting with 1,000 ms of stimulus presentation, followed by a fixation of 750 ms, then a cue for response or a fixation for 2250 ms, and ended with a fixation of 2000 ms (Fig 1, row C). The participants’ pronunciations were recorded online with a MR-compatible microphone (FOM1-MR model provided by Micro Optics Technologies, Inc.).

### Sound recording analysis

Consulting the sound recordings and their spectrograms, trials with mispronunciation or 33 trials without Tone 3 sandhi were identified by the authors and excluded from further acoustic and imaging analyses. Six of the thirty participants were excluded for high rejection rate. Among them, one always pronounced the stimulus twice even in the monosyllable sessions and five applied Tone 3 sandhi far rarely (5%, 5%, 5%, 15%, and 35% out of all the 33 trials with overt oral response) than the remaining twenty-four participants (mean 92%, range: 80%-100%). The distinctive difference between these two groups of participants might result from the usage of the phonetic symbols, i.e. Zuyin. We used phonetic symbols rather than character to diminish lexical processing. However, since Zuyin is often used to teach pronunciation of characters to elementary students or to correct mispronunciation, some of the participants might make extra efforts to match their pronunciation with the denoted sound and thus interrupted the implementation of Tone 3 sandhi. This explanation is supported by studies using character [40] or auditory stimuli [41,42], which reported a high proportion of Tone 3 sandhi application as in our remaining twenty four participants, while one study including both Chinese characters and phonetic symbols reported lower rate of Tone 3 sandhi application for stimuli presented in phonetic symbols [42].

Although the sound recordings were clean enough to identify mispronunciations for all the participants, to display the full pitch contours, a more stringent requirement for recording quality is necessary. We excluded trials with any acoustic disturbance and participants with too many disturbed trials from acoustic analysis. Among the twenty-four participants whose imaging data were included in the fMRI analysis, sound recordings from seventeen of them were pooled together to demonstrate the pitch contours of the four tones under the monosyllable and disyllable conditions. The acoustic disturbance often came from airflow produced during speech and the scanner noise. Although we have used the sparse sampling technique to enable a 1200 ms silent period 50 ms after stimulus presentation for response recording, with long reaction time or long duration of pronunciation, part of the second syllable in a disyllable sequence could still be contaminated by the scanner noise.

The sound recordings were processed using the software Praat [43] and the program ProsodyPro [44]. Time-normalization was done by taking 16 points from each syllable at equal proportional intervals. Speaker-normalization was done through division by the speaker’ pitch ranges (highest pitch—lowest pitch) after subtracting the midpoint of the pitch range ((highest pitch + lowest pitch)/2).

### MRI acquisition

MR scanning was performed using a 3T MRI (Tim Trio, Siemens, Erlangen, Germany) interfaced with a 32-channel phased-array head coil. A T2*-weighted gradient-echo echo planar imaging (EPI) sequence was used for fMRI scanning. To provide a short silent period for sound recording, the sparse sampling acquisition for the EPI images was arranged (Eden et al., 1999; Edmister et al., 1999; Hall et al., 1999) with the slice thickness = 3.4 mm, in-plane resolution (64 x 64) = 3.4 x 3.44 mm, and TR/TE/θ = 3,000 ms/30 ms/90°. The delay in TR was 1,200 ms after each volume acquisition. Thirty-three axial slices were acquired to cover the whole-brain. There were 200 repetitions in an EPI session. The anatomical, T1-weighted high-resolution image (1 x 1 x 1 mm) was acquired using a standard MPRAGE sequence (TR/TE/TI = 2,530/3.49/1,100 ms, flip angle = 7°). The total duration of the fMRI experiment was about 40 minutes.

### MRI data analysis

Data processing was performed with SPM8 (Wellcome Department of Cognitive Neurology; software available at http://www.fil.ion.ucl.ac.uk/spm). Functional images were corrected for slice timing, head motion, normalized to the avg152 T1-weighted brain template defined by the Montreal Neurological Institute, and spatially smoothed with an isotropic Gaussian filter (8 mm full width at half maximum).

In the first-level SPM model, experimental effects at each voxel were estimated using a multi-session design matrix modeling the 16 conditions (monosyllable/disyllable x overt/no oral response x four tones), the baseline condition, trials with erroneous response, and six movement parameters. The regressors were obtained by convolving the impulse response with the canonical SPM hemodynamic response function, its time derivative, and its dispersion derivative. Contrasts between each of the 16 experimental conditions and the baseline condition were computed.

These estimates of the individual effect sizes were entered into a second-level analysis with one regressor for each condition, as well as for each participant. Test examining the 2 x 2 x 4 interaction between overt-response, number of syllable, and tone was conducted and no significant effect was found. However, the test of the 2 x 2 x 4 interaction is to examine whether the difference between the four tones was dependent on overt oral response and number of syllable, while our a priori hypothesis is that Tone 3 sandhi requires additional processing, which would result in larger brain responses for Tone 3 than the other tones. Therefore, instead of differentiating the four tones, we contrasted Tone 3 with the other tones and examined the 2 x 2 x 2 interaction (Overt/No response X Monosyllable/Disyllable X Tone3/Others). We first search for regions showing the three-way interaction (from Tone 1, Tone 2, Tone 3, to Tone 4, the contrast weights were -1/3, -1/3, 1, -1/3 for disyllable overt response condition and monosyllable no response condition, while the contrast weights were 1/3, 1/3, -1, 1/3 for disyllable no response condition and monosyllable overt response condition). Activations were thresholded at p < .05 and corrected at cluster level with FWE corrected p < .05. Then we contrasted Tone 3 with the other tones under the four conditions: monosyllable/overt oral response, monosyllable/no oral response, disyllable/overt oral response, and disyllable/no oral response. The p-values of the four tests were adjusted to .0125 (= .05/4) for the number of tests using the Bonferroni method. From Tone 1, Tone 2, Tone 3, to Tone 4, the contrast weights were -1/3, -1/3, 1, -1/3.

For clusters showing significant activations, local peaks were identified and labeled using the AAL tool box (Tzourio-Mazoyer et al., 2002). All coordinates were reported in MNI coordinate space.

### ROI analysis

To perform region of interest (ROI) analyses, we generated masks for anatomical regions known to be involved in speech production from the AAL ROI archive [45], including middle frontal gyrus (MFG), the opercular, triangular, and orbital parts of IFG, anterior precentral gyrus, postcentral gyrus, supplementary motor area (SMA), STG, thalamas, putamen, and anterior insula (the part of insula anterior to y = 0). Our criterion of ROI selection is independent of the effect in interest, and thus the problem of circularity was avoided [46,47]

A repeated measure 3-way ANOVA was performed for each region, with a factor for Tone 3 (1 for Tone 3, 0 for the other tones), one factor for number of syllable, and one for overt oral response. Generalized eta-squared (η^2^) [48] and Cohen’s d [49] were reported as measures of effect size for ANOVA and t-test respectively.

## Results

### Behavioral results

**Fig 2:**
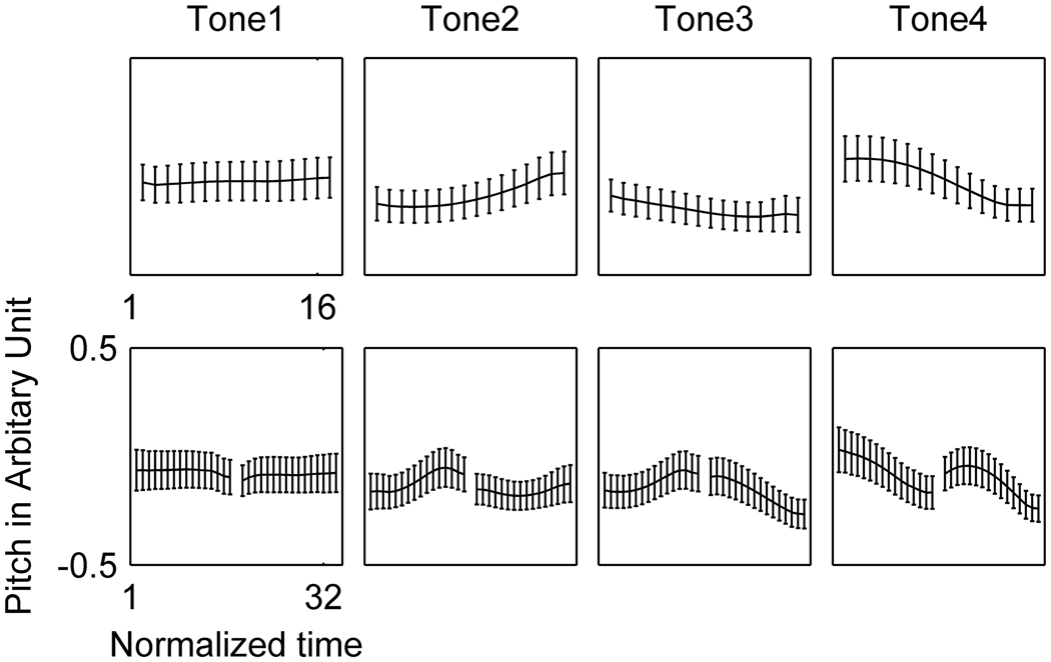
Pitch contours of the four lexical tones under the monosyllable (upper row) and disyllable conditions (bottom row). 16 points from each syllable at equal proportional intervals were taken and their values were divided by individual speaker’s pitch range after subtracting the midpoint of the pitch range. Error bars represent ± 1 SE between speakers. The Tone 3 sandhi was manifested by the rising contour of the initial Tone 3 in the 33 sequence, which was similar to Tone 2, but different from monosyllable Tone 3 and Tone 3 at the final position of sequence 33.

The averaged mispronunciation rate for trials with overt oral response was 3% (range: 0%-12%). The averaged pitch contours of the four tones under monosyllable and disyllable conditions are displayed in Fig 2. Tone 3 sandhi is manifested by the initial Tone 3 of sequence 33, whose rising contour was more similar to Tone 2 than to the low-falling contour of monosyllable Tone 3 or Tone 3 at the final position of sequence 33. It is worth mentioning that the low-falling contour of monosyllable Tone 3 in our results is different from the falling-rising pattern in standard Mandarin [40], but consistent with previous studies in Taiwanese Mandarin [50,51], which might reflect the influence from Taiwanese dialect [52].

### fMRI results

We hypothesized that Tone 3 sandhi requires additional processing, which would be reflected by higher brain activations for Tone 3 than the other tones. Since Tone 3 sandhi only applies to multi-syllable Tone 3 sequence, such effect was expected only under the disyllable condition, not the monosyllable condition. Namely, we predict a two-way interaction between number of syllable and the Tone 3 effect. Further, we are interested in whether such effect would be dependent on overt production. If so, a 2 x 2 x 2 three-way interaction should be observed.

#### Results of whole-brain analysis

**Fig 3:**
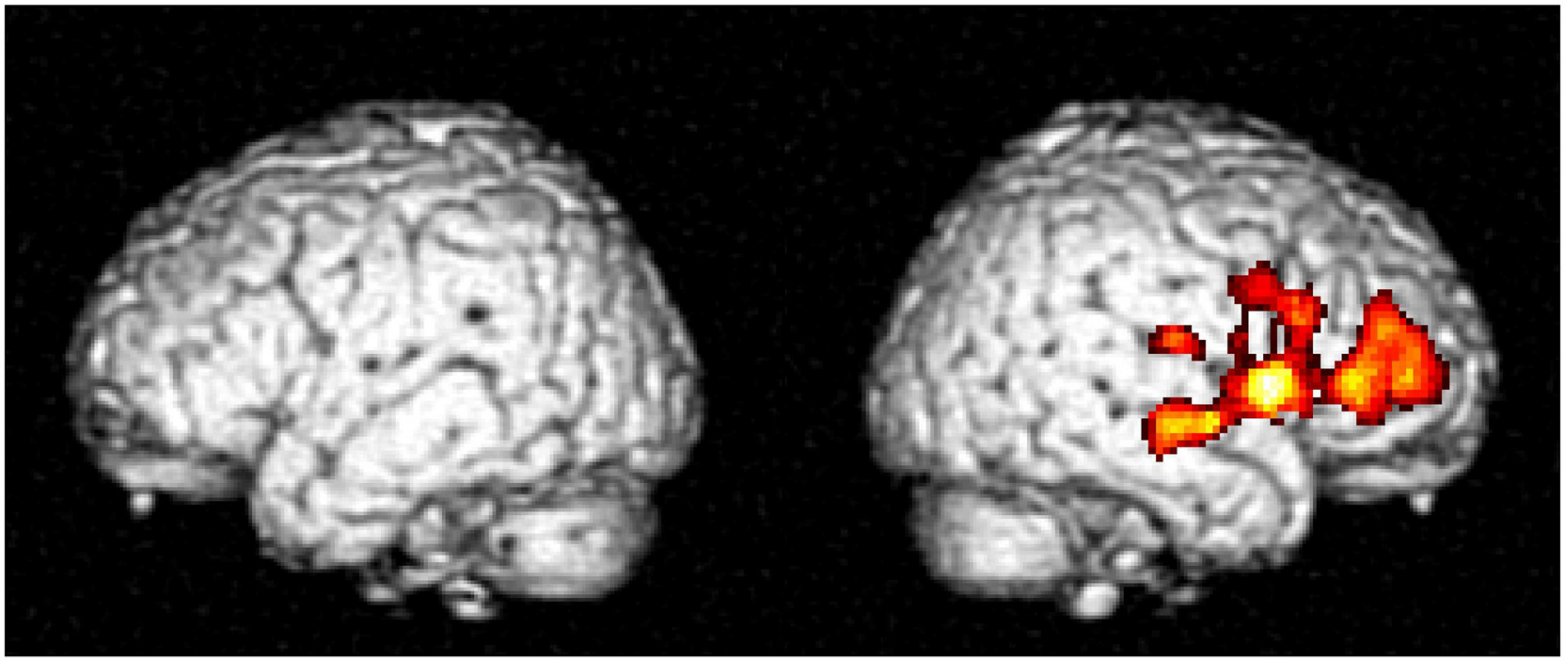
SPMt map for brain regions showing the three-way interaction between overt oral response, number of syllable, and the Tone 3 effect (N = 24; voxelwise uncorrected threshold of p < .05 and clusterwise FWE corrected threshold of p < .05).

Three-way interaction between overt oral response, number of syllable, and the Tone 3 effect was revealed at a low voxelwise threshold (p < .05) with cluster-level correction (FWE < .05), which indicates a small but consistent effect within the cluster. Local peaks within the cluster were found in the right middle and inferior frontal gyrus, right insula, right STG, and subcortical regions including right putamen (Fig 3 and Table 1).

**Table 1:** Interaction between overt oral response, number of syllable, and the Tone 3 effect.

Region	Hemisphere	T-value	X	Y	Z
Middle frontal gyrus	Right	2.53	40	52	12
Inferior frontal gyrus pars triangularis	Right	2.71	50	34	4
Inferior frontal gyrus pars opercularis	Right	3.00	56	14	4
Insula	Right	2.85	40	-4	-2
Superior temporal gyrus	Right	2.52	56	-12	-8
Rolandic operculum	Right	2.67	46	-18	18
Putamen	Right	3.09	30	-6	0
Globus pallidus	Right	2.92	28	-10	0

**Fig 4:**
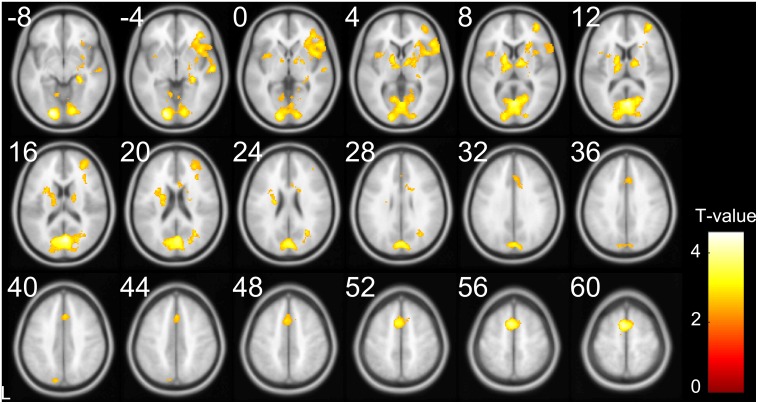
SPMt map for regions showing larger responses to disyllable sequences of Tone 3 than the other tones (33 > 11, 22, 44) under overt oral response condition.

To test the a priori hypothesis that Tone 3 sandhi requires additional processing, which would result in larger brain responses to Tone 3 than the other tones, four contrasts were conducted. We contrasted Tone 3 with the other tones (Tone 3 vs. 124) under the four conditions: monosyllable/overt oral response, monosyllable/no oral response, disyllable/overt oral response, and disyllable/no oral response. Only under one condition—i.e., the overt disyllable condition—was a significant effect observed (Fig 4 and Table 2). Larger activations for Tone 3 were found in the right IFG, right MFG, right insula, left SMA, right superior temporal gyrus, bilateral subcortical regions, and bilateral visual areas. To further verify our findings, contrasts for the other three tones arranged in the same way (Tone 1 vs. 234; Tone 2 vs. 134; Tone 4 vs. 123) were also examined and except in the visual areas, no positive effect was found under any conditions.

**Table 2:** 33 > (11, 22, 44) effect under the overt oral response condition.

Region	Hemisphere	T-value	X	Y	Z
Middle frontal gyrus	Right	3.13	38	50	8
Inferior frontal gyrus pars triangularis	Right	3.29	52	24	2
Inferior frontal gyrus pars opercularis	Right	3.02	56	16	4
Insula	Right	3.14	38	14	-2
Supplementary motor area	Left	4.16	-4	6	60
Superior temporal gyrus	Right	3.30	60	-16	-4
Hippocampus	Right	3.38	28	-30	-4
Cuneus	Left	3.55	-16	-78	16
Middle occipital gyrus	Left	4.58	-16	-88	-6
Lingual gyrus	Right	3.59	14	-84	-6
Calcarine sulcus	Left	4.06	-4	-74	14
Calcarine sulcus	Right	4.43	4	-72	14
Globus pallidus	Left	3.70	-14	0	6
Thalamus	Left	3.20	-14	-16	10
Thalamus	Right	3.61	12	-8	8

**Fig 5:**
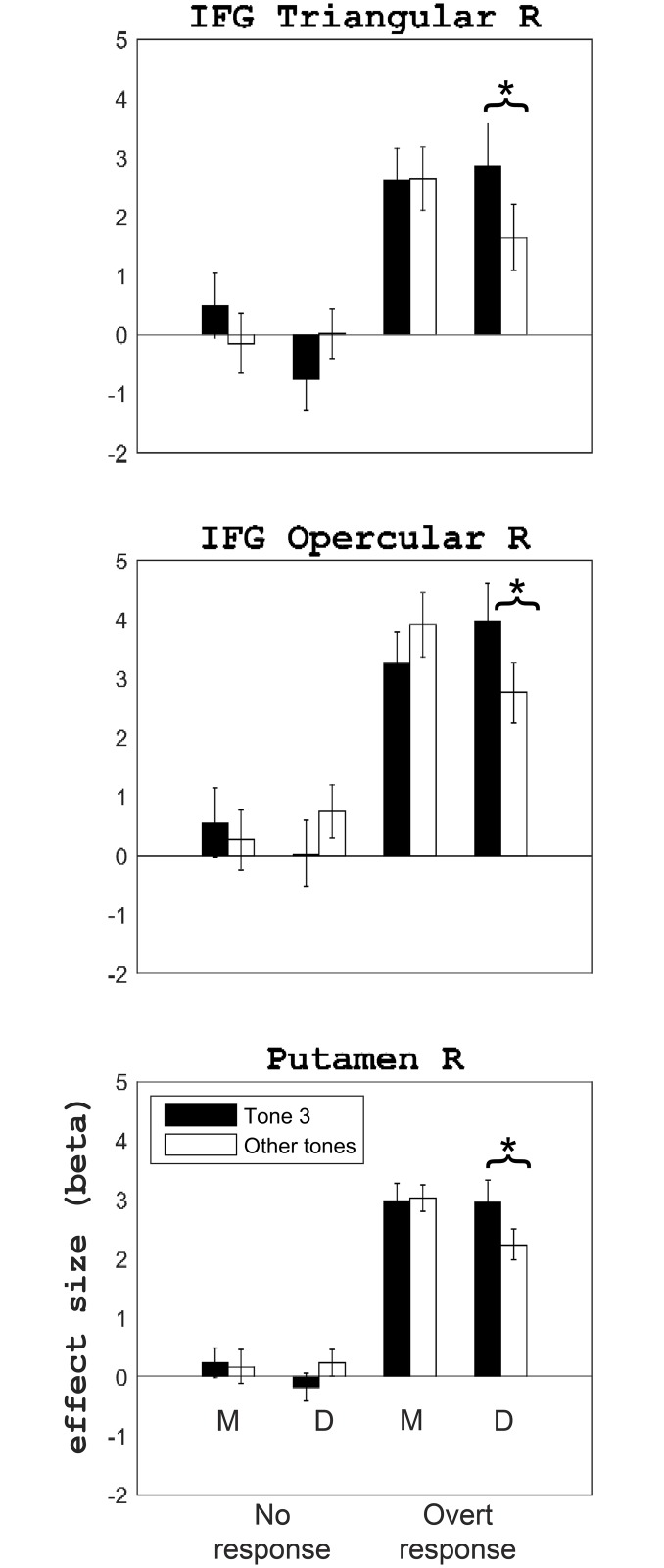
Averaged fMRI responses to Tone 3 and the other tones within anatomically defined brain regions showing significant interaction between the Tone 3 effect, number of syllable, and overt oral response in the ROI analysis (p < .05, Bonferroni correction, N = 24). Error bars represent 1 SEM of the responses after removing the main effect of participant. M: monosyllable condition. D: disyllable condition. The asterisks denote significant differences between Tone 3 and the other tones in the post-hoc t-tests.

Negative effect between Tone 1 and the other tones (234 vs. 1) in the visual areas was found under all conditions, which was presumably resulted from the fact that Tone 1 was denoted by the absence of tonal symbols, while all the other tones had corresponding tonal symbols (Fig 1A). The occipital cluster shown in Fig 5 was also a result of the lower activations for Tone 1, which was clearly demonstrated by examining the activation patterns at the peak of activations in the visual cortex ([–16, –88, –6], S1 Fig)

#### Results of ROI analysis

The ROI analysis revealed a three-way interactions between number of syllable, overt oral response, and the Tone 3 effect in right IFG triangular (η^2^ = .01, F(1,23) = 6.38, p = .020), right IFG opercular (η^2^ = .01, F(1,23) = 7.42, p = .012), and right putamen (η^2^ = .009, F(1,23) = 6.54, p = .010). These regions also showed the three-way interaction in the whole-brain analysis. Significant two-way interaction between number of syllable and the Tone 3 effect was not observed in any ROIs.

Fig 5 indicates that the three-way interaction reflected the predicted interaction between number of syllable and the Tone 3 effect, and that such effect was dependent on overt oral response. In other words, larger brain activation for Tone 3 was only found under disyllable condition and when overt oral response was required (for averaged brain activations to the four tones within all ROIs under each condition, please see S4 Fig).

To validate this interpretation, for regions showing the three-way interaction, paired one-tailed t-test between Tone 3 and the other tones were performed under the following four conditions: monosyllable/overt oral response, monosyllable/no oral response, disyllable/overt oral response, and disyllable/no oral response (p-value was adjusted for the number of tests using the Bonferroni method). For all three regions, significantly larger responses to Tone 3 was found only under disyllable overt oral response condition (right IFG triangular: Cohen’s d = 0.49, t(23) = 2.42, p = .048; right IFG opercular: Cohen’s d = 0.53, t(23) = 2.58, p = .034; right putamen: Cohen’s d = 0.61, t(23) = 2.97, p = .014) (Fig 5).

In summary, both the whole-brain analysis the and ROI analysis revealed three-way interaction between number of syllable, overt oral response, and the Tone 3 effect in IFG triangular, IFG opercular, and putamen. Post-hoc analysis showed that the three-way interaction reflected larger brain responses to Tone 3 than the other tones under the overt disyllable condition.

## Discussion

This study aims to investigate the neural substrates underlying phonological rule in lexical tone. We adopted a tone production task which triggered Tone 3 sandhi. Tone 3 sandhi implicitly substitutes the tone representation to be articulated. The experiment included three factors: tone, number of syllable, and overt oral response. We expected disyllable Tone 3 to elicit Tone 3 sandhi, which was confirmed by our behavioral results. To reveal the brain activations corresponding to the processing of Tone 3 sandhi, we contrasted Tone 3 with the other tones and found larger brain activations for Tone 3 only under overt disyllable condition (33 > 11, 22, 44) in right IFG, right MFG, right insula, left SMA, right STG, and bilateral subcortical regions. Consistent with the whole-brain analysis, the ROI analysis also showed that the Tone 3 effect interacted with syllable number and overt oral response in the opercular and triangular parts of right IFG, as well as right putamen.

The fact that larger brain activations for Tone 3 only arose under the disyllable condition but not the monosyllable condition verified that our findings did not only result from some inherent physical difficulty of producing Tone 3. However, because of Tone 3 sandhi, sequence 33 was pronounced as mixed sequence 23 on the surface. Mixed sequence could increase the processing loading on retrieval and sequencing. Mixed sequence might also require extra computation for coarticulation and change of pitch direction [38,53]. Is it possible that the larger activations for disyllable Tone 3 only reflected the processing of mixed sequence? A recent ERP study directly compared the covert production of tone sequence 23 and 33. In spite of that they were both pronounced as 23 on the surface, it was found that sequence 33 elicited larger P2 than 23 [42], supporting that Tone 3 sandhi does require additional processing. Further, in our previous study [31], the neural correlates underlying “genuine” mixed sequences (twelve of them, e.g. 2413, 1324, etc.) and sequence 3333 were examined respectively, taking repeated sequences as baseline (1111, 2222, and 4444). Genuine mixed sequence also showed increased activations in the left SMA, bilateral insula, and bilateral putamen, but activations in the right posterior IFG and right anterior MFG were only observed in sequence of Tone 3.

The right IFG showed higher activations for Tone 3 sequence in both our whole-brain and ROI analyses, but not for genuine mixed sequence or monosyllable Tone 3. We suggest that the right IFG was responsible for the processing of Tone 3 sandhi. The connection between the right IFG and the right superior temporal lobe was reported to correlate with the performance in learning pitch-based grammar rule [24]. Further, dysfunction [54] or disconnection of the right IFG with the auditory cortex through arcuate fasciculus caused congenital amusia [55], an impairment to process music melody as well as lexical tone [56–59]. The right IFG could be recruited in tone processing through its interaction with the right auditory cortex, which is known to specialize in pitch [7–11]. During speech production, intense interaction between the frontal and temporal regions are necessary for self-monitoring and error correction [6,29,37], namely, to check whether the auditory feedback matches the expected output and adjust the motor command accordingly. The specialization of the right auditory cortex in pitch might influence the right inferior frontal regions through their interaction via arcuate fasciculus [25,26]. Namely, there might be a right temporo-frontal pathway parallel to the known left language pathway. Similar dual pathway model has been proposed for sentence level prosody [60].

A The right MFG was associated with auditory selective attention according to meta-analysis of imaging studies in language [61]. Consistent with this interpretation, right MFG has also been reported in the categorization of non-speech pitch [62] and discrimination of sounds with degraded spectral information [63]. As explained in the first paragraph of section 2.3, although Tone 3 sandhi is an automatic process and native-speakers usually are not aware of it, in the experimental setting, the participants might have put additional effort in matching their pronunciations with the displayed phonetic symbols. With the application of Tone 3 sandhi, the discrepancy between the underlying and the surface tones could lead to selective attention to pitch, thus the increased activations in right MFG in the whole-brain analysis.

In our current and previous [31] studies, SMA, anterior insula, and putamen were found to be engaged in the processing of mixed sequences, no matter they were “genuine” or derived. These regions are parts of the established language network [5,32,64], which are involved in the production of both tone and non-tone languages. Higher activations in left SMA has also been reported for mixed sequences of syllables [65] and movements [66,67], consistent with neurophysiological study showing that cells in SMA proper responded selectively to the initiation of movement sequences and cells in pre-SMA were sensitive to transition between certain movement pairs in sequences [68]. The left SMA might be responsible for the sequencing of the motor targets at the planning stage of articulation, while the anterior insula and the putamen are more for the execution. Insula and putamen have been found to form a functional network with the motor cortex, inferior cerebellum, and other subcortical areas in speech production. Peak activations of regions within this network occurred seconds after the peak activation of left SMA [69]. Lesion in the left anterior insula was associated with impairment in coordinating articulators during speech [70,71]. Imagining studies suggested that the right anterior insula is also involved in speech processing [72]. There are studies showing that speech and singing elicited opposite lateralization patterns in the anterior insula [33]. The fact that both melody and lexical tone are mediated by pitch patterns might explain why we found right-lateralized activations in the anterior insula. On the other hand, putamen was suggested to participate in speech tempo control, based on studies in dysarthria and imaging studies showing that activations in putamen was dependent on the rhythm of speech [73–75].

We manipulated the requirement on overt oral response in order to distinguish pre-articulatory planning and motor execution stages of speech production. Following our original idea, any effects found only under the overt oral response condition would be interpreted as dependent on motor execution. Indeed, even without overt oral response, the language network was partially activated (S2 Fig). However, even the number of syllable effect, which has been shown to be independent of motor execution through covert speech task [76,77], was not observed without overt oral response in our experiment (S3 Fig). The difference between ours and previous studies may come from the fact that we did not ask our participants to pronounce the stimuli covertly. After all, people do not rehearse consciously before speaking in real life. We assumed that the pre-articulatory planning stage would initiate upon the presentation of the stimuli, since overt oral response was required in half of the trials. This discrepancy between studies highlights the problem of using tasks instead of measurement with better temporal resolution to differentiate stages of processing. That the planning for disyllable sequence was not initiated could well be the reason why Tone 3 sandhi effect was not observed without overt oral response, since Tone 3 sandhi only applies to sequence of Tone 3. In addition, according to a recent study using covert production task, Tone 3 sandhi effect is not necessarily dependent on motor execution [42]. For the above reasons, although Tone 3 sandhi effect was only found under the overt oral response condition in our experiment, it is better not to jump to the conclusion that Tone 3 sandhi is dependent on motor execution. To solve this issue, using tools with better temporal resolution is necessary.

It is worth noticing that using speech production task and the adaptation paradigm, the contrast between monosyllable tone and vowel has also revealed activations in the right IFG and right insula, although their contralateral parts still showed larger activations [78]. Therefore, our results do not indicate that the right IFG is only involved when tone sandhi is applied. Instead, the interaction within and between the dual temporo-frontal pathways depends on the loading on phonological processing and the concurrent segmental information. Increased right IFG activations for Tone 3 sequence could result from that Tone 3 sandhi involved extra processing of tone representation but no additional processing for segment. With the limited temporal resolution of fMRI, it is difficult to examine the dynamics of the interaction between the two pathways. We can only speculate that if the right IFG is responsible for the processing of Tone 3 sandhi and left SMA for the motor planning of the derived mixed sequence, then the output of the right temporo-frontal pathway must converge to the left hemisphere for further processing. The left SMA might be responsible for the sequencing and alignment of both tone and segment to enable by-syllable speech production [79]. This scenario is compatible with studies showing that the left hemisphere is dominant for bimanual coordination [80,81] and the sequencing of movements executed by either hand [82,83]. Still, future studies using tools with better temporal resolution are needed to clarify the neural dynamics between the two hemispheres in tone languages [84].

## Supporting Information

S1 Fig Estimated effect size for the four tones at [–16, –88, –6] under all conditions. Error bars represent 90% CI.
(DOCX)

S2 Fig SPMt maps for the monosyllable and disyllable conditions with and without overt oral response respectively. (DOCX)

S3 Fig SPMt map for brain regions showing larger responses under disyllable then monosyllable condition (disyllable > monosyllable) in trials with and without overt oral response respectively. (DOCX)

S4 Fig Averaged fMRI responses to the four tones within all regions of interest under each condition. (DOC)
