# Supplementary figures and images for: The Neural Substrates Underlying the Implementation of Phonological Rule in Lexical Tone Production: An fMRI Study of the Tone 3 Sandhi Phenomenon in Mandarin Chinese
Source: PLoS One. 2016 Jul 25;11(7):e0159835. doi: 10.1371/journal.pone.0159835 (PMC4959711; doi:10.1371/journal.pone.0159835)

S1 Fig. Estimated effect size for the four tones at [-16, -88, -6] under all conditions. Error bars represent 90% CI.


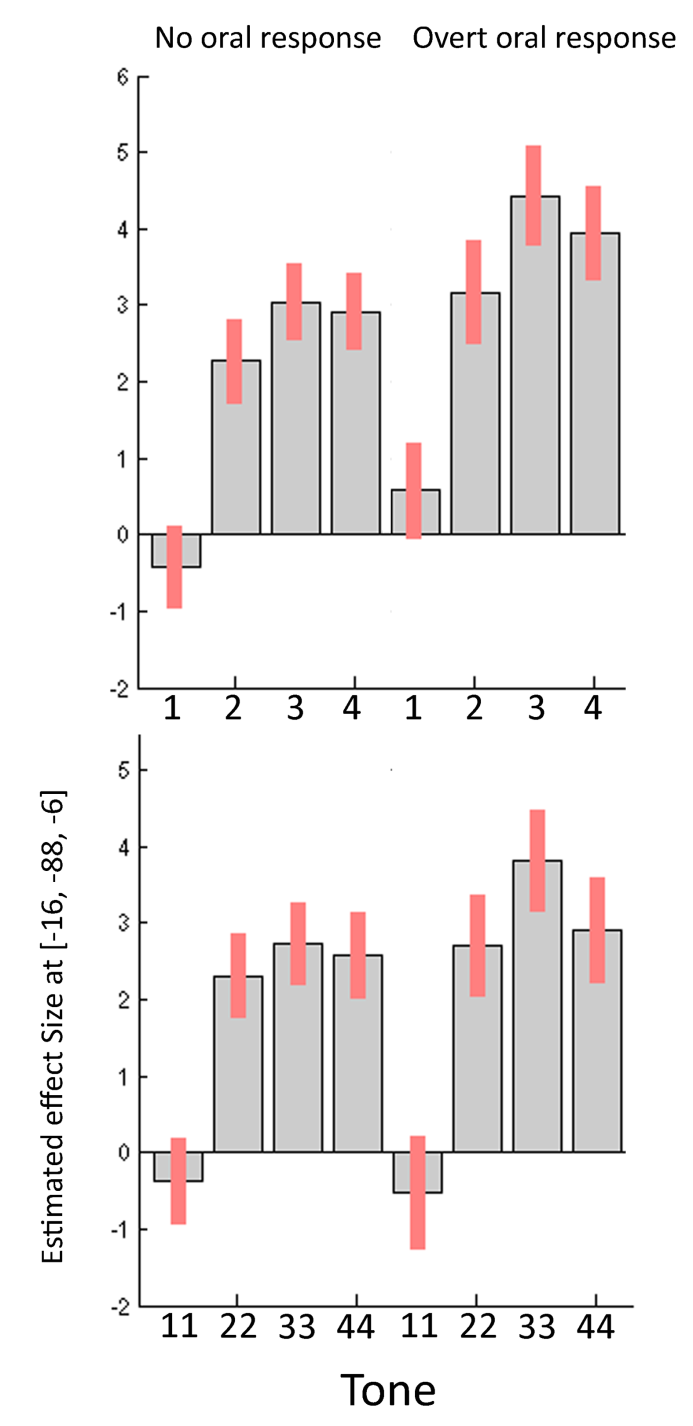

Supplement: S1 Fig — Error bars represent 90% CI. (DOCX) [file pone.0159835.s001.docx]
