# Supplementary material for: The Neural Substrates Underlying the Implementation of Phonological Rule in Lexical Tone Production: An fMRI Study of the Tone 3 Sandhi Phenomenon in Mandarin Chinese
Source: PLoS One. 2016 Jul 25;11(7):e0159835. doi: 10.1371/journal.pone.0159835 (PMC4959711; doi:10.1371/journal.pone.0159835)

S2 Fig. SPMt maps for the monosyllable and disyllable conditions with and without overt oral response respectively.


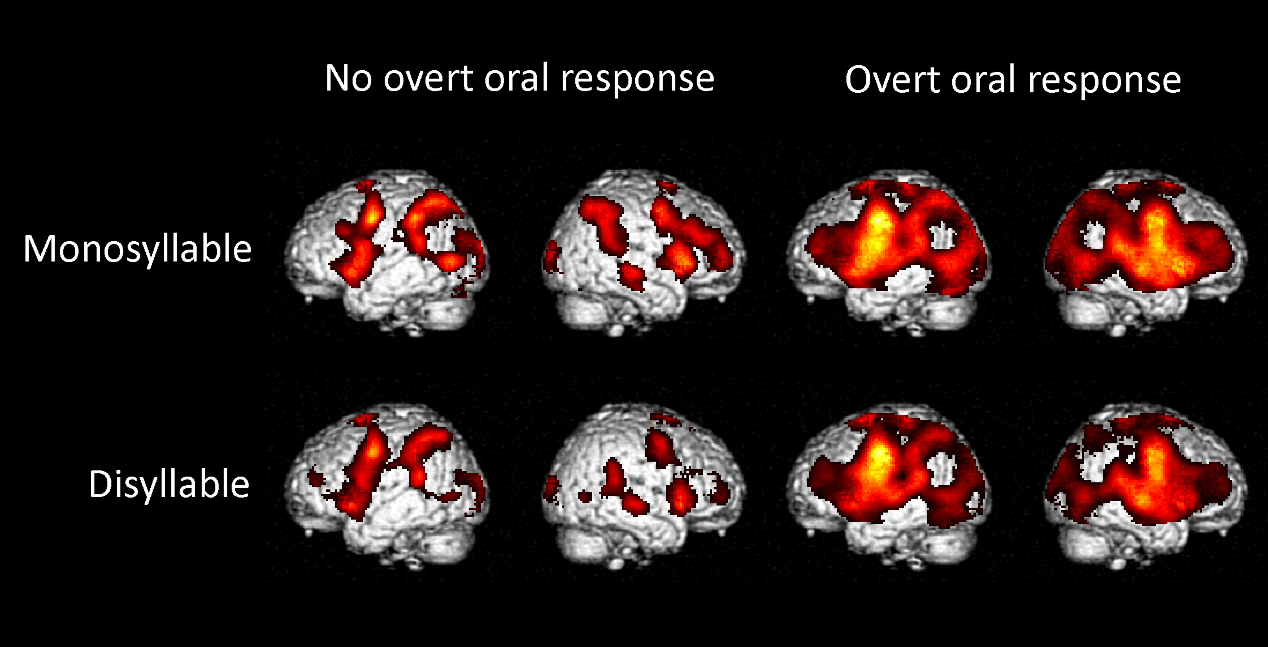

Supplement: S2 Fig — (DOCX) [file pone.0159835.s002.docx]
