# Supplementary material for: The Neural Substrates Underlying the Implementation of Phonological Rule in Lexical Tone Production: An fMRI Study of the Tone 3 Sandhi Phenomenon in Mandarin Chinese
Source: PLoS One. 2016 Jul 25;11(7):e0159835. doi: 10.1371/journal.pone.0159835 (PMC4959711; doi:10.1371/journal.pone.0159835)
Supplement: S3 Fig — (DOCX) [file pone.0159835.s003.docx]

S3 Fig. SPMt map for brain regions showing larger responses under disyllable then monosyllable condition (disyllable > monosyllable) in trials with (right) and without (left) overt oral response respectively (N = 24; voxelwise uncorrected threshold of p < .01 and clusterwise FWE corrected threshold of p < .05. The same threshold was used throughout this study unless otherwise stated).


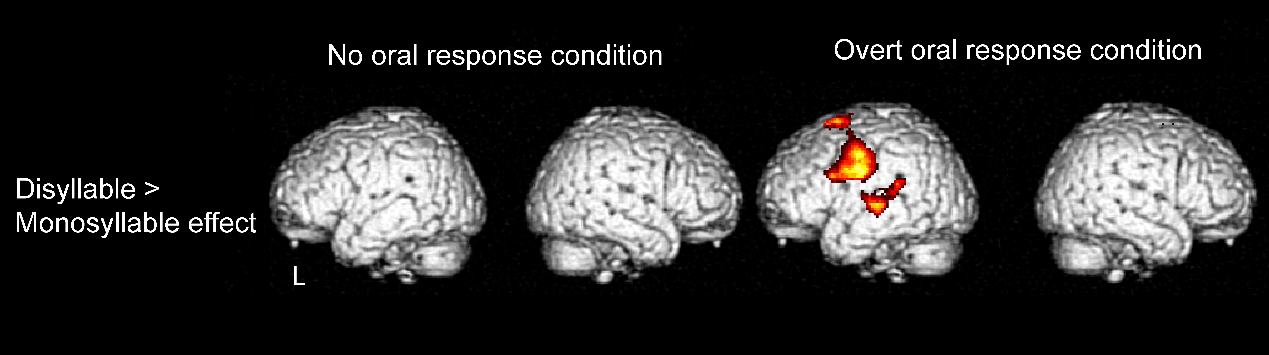


The comparison between disyllable and monosyllable conditions were conducted under the over oral response and no response conditions separately. With overt oral response, higher activations under disyllable than monosyllable condition were found in bilateral SMA, left precentral gyrus, left STG, and left middle temporal gyrus (MTG). No such effect was found when oral response was not executed.
