# Supplementary material for: The Neural Substrates Underlying the Implementation of Phonological Rule in Lexical Tone Production: An fMRI Study of the Tone 3 Sandhi Phenomenon in Mandarin Chinese
Source: PLoS One. 2016 Jul 25;11(7):e0159835. doi: 10.1371/journal.pone.0159835 (PMC4959711; doi:10.1371/journal.pone.0159835)
Supplement: S4 Fig — (DOC) [file pone.0159835.s004.doc]

S3 Fig. 1 Averaged fMRI responses within anatomically-defined ROIs to the four tones under monosyllable condition with overt oral response. Error bars represent 1 SEM of the responses after removing the main effect of participant (N = 24).





S3 Fig. 2 Averaged fMRI responses within anatomically-defined ROIs to the four tones under disyllable condition with overt oral response. Error bars represent 1 SEM of the responses after removing the main effect of participant (N = 24).





S3 Fig. 3 Averaged fMRI responses within anatomically-defined ROIs to the four tones under monosyllable condition when no overt oral response was executed. Error bars represent 1 SEM of the responses after removing the main effect of participant (N = 24).





S3 Fig. 4 Averaged fMRI responses within anatomically-defined ROIs to the four tones under disyllable condition when no overt oral response was executed. Error bars represent 1 SEM of the responses after removing the main effect of participant (N = 24).
